# COGNITIVE RESERVE AND DISPARITIES IN HEALTHCARE USAGE AFTER TRAUMATIC BRAIN INJURY AND STROKE: AN OBSERVATIONAL COHORT STUDY

**DOI:** 10.2340/jrm.v57.42400

**Published:** 2025-05-13

**Authors:** Natascha EKDAHL, Marianne LANNSJÖ, Britt-Marie STÅLNACKE, Marika C. MÖLLER

**Affiliations:** 1 Department of Clinical Sciences, Danderyd Hospital, Karolinska Institutet, Stockholm; 2 Centre for Research and Development, Uppsala University/County Council of Gävleborg, Gävle; 3 Faculty of Health and Occupational Studies, University of Gavle, Gävle; 4 Department of Medical Sciences, Rehabilitation Medicine, Uppsala University, Uppsala; 5 Department of Community Medicine and Rehabilitation, Rehabilitation Medicine, Umeå University, Umeå; 6 Department of Rehabilitation Medicine, Danderyd University Hospital, Stockholm, Sweden

**Keywords:** brain injuries, stroke, educational status, delivery of healthcare

## Abstract

**Background:**

Individuals with more education commonly have better outcome after brain injury, often attributed to cognitive reserve. However, evidence suggests that individuals with more education have better access to specialized care, potentially affecting outcomes.

**Objective:**

To investigate differences in healthcare usage based on cognitive reserve and examine the relationship between healthcare usage and outcomes after stroke and traumatic brain injury.

**Design:**

An observational cohort study with healthcare usage data from 3 years before to 4 years after injury, interviewing patients 5–15 years after injury.

**Patients:**

A total of 83 participants suffering a stroke or traumatic brain injury.

**Results:**

Healthcare usage over time varied based on educational level (repeated measures ANOVA, F(2, 227) = 4.17, *p* = 0.008). The differences in healthcare usage between educational levels was significant during the injury year (F(81) = –5.47, *p* = 0.022). Higher education implied more healthcare usage. Linear regression, controlling for possible confounders, confirmed the relationship between education and healthcare usage, (β = 4.3, *p* = 0.022). Healthcare usage was significantly related to long-term life satisfaction, but not to return to work.

**Conclusion:**

Individuals with more education received more healthcare in the year after traumatic brain injury or stroke. However, this was not related to long-term outcome regarding return to work, but we found a relationship between healthcare usage and life satisfaction.

## LAY ABSTRACT

People with higher education levels usually have better outcomes after brain injuries, which is often linked to cognitive reserve. However, they might also have better access to specialized care, influencing these outcomes. This study aimed to explore how healthcare usage varies with cognitive reserve and its impact on recovery after stroke and traumatic brain injury. We conducted an observational study with 83 participants, analysing healthcare usage from 3 years before to 4 years after their injury and interviewing them 5–15 years post-injury. Results showed that those with higher education used more healthcare services, especially in the year of the injury. This increased usage was linked to higher life satisfaction but not to the likelihood of returning to work. In conclusion, while more educated individuals received more healthcare, healthcare usage was not related to better long-term work outcomes, though possibly to life satisfaction.

After traumatic brain injury (TBI) or stroke, there is an increased need for healthcare (1, 2). However, this increased demand may not be evenly distributed (3). People with lower income tend to have more health issues and in general consume more health resources, but when controlling for health status people with higher socioeconomic status tend to use more specialized healthcare (4). Those with lower socioeconomic status rely more on general practitioners, even in countries with generous welfare systems (4, 5). This disparity exists in the general population and among people with specific conditions, such as spinal cord injury and heart disease (6–8). Better access to healthcare leads to timely and appropriate interventions, improving general health (9, 10). It is plausible that better access to specialized healthcare is also linked to better outcomes after acquired brain injury.

It is recognized that educational level is associated with outcomes after brain injury. Individuals with higher education tend to have better cognitive, return-to-work, and psychological outcomes (11, 12). This has been attributed to cognitive reserve – the concept that people with higher education have more cognitive resources that can be used to compensate for brain injury, thereby lessening the impact (13). However, higher education correlates with higher socioeconomic status, leading to more specialized healthcare use. Hence, an uneven distribution in the utilization of specialized healthcare after brain injury, based on educational level, might explain some of the better outcomes in people with higher education.

Some American studies have found a correlation between lack of insurance, which often accompanies lower socioeconomic status, and reduced utilization of healthcare following acquired brain injury (14, 15). A Swedish study on TBI patients found that higher educational levels were linked to receiving specialized rehabilitation. This study did not examine the effect of education on other types of healthcare usage, or the amount of rehabilitation received (16). Given this context, our study aimed to investigate whether there were any differences in healthcare usage, both in total and for inpatient, outpatient, and primary care separately, related to cognitive reserve, measured by educational level, after stroke or TBI. We also aimed to investigate whether return-to-work and life satisfaction after stroke and TBI was related to healthcare usage, thereby providing a deeper understanding of how educational level, as a proxy for cognitive reserve, is related to outcome after brain injury.

## Study setting

An observational cohort study was conducted at a Rehabilitation Medicine Clinic in a central Swedish region with about 300,000 inhabitants. A specialized outpatient brain injury rehabilitation team provides multidisciplinary team rehabilitation for working-age adults with acquired brain injuries, supporting both patients and their significant others in everyday life and work rehabilitation. Ethics approval was obtained from the Regional Ethical Review Board in Uppsala, diary no: 2018/242, 2020-05887, 2021-02002.

The Brain Injury Rehabilitation Team includes a physician specialist in rehabilitation medicine, an occupational therapist, a social worker, and a psychologist. Average duration of intervention is 2.5 years, frequency of contact varying between every week to every 3 months.

## Participants

**Fig. 1:**
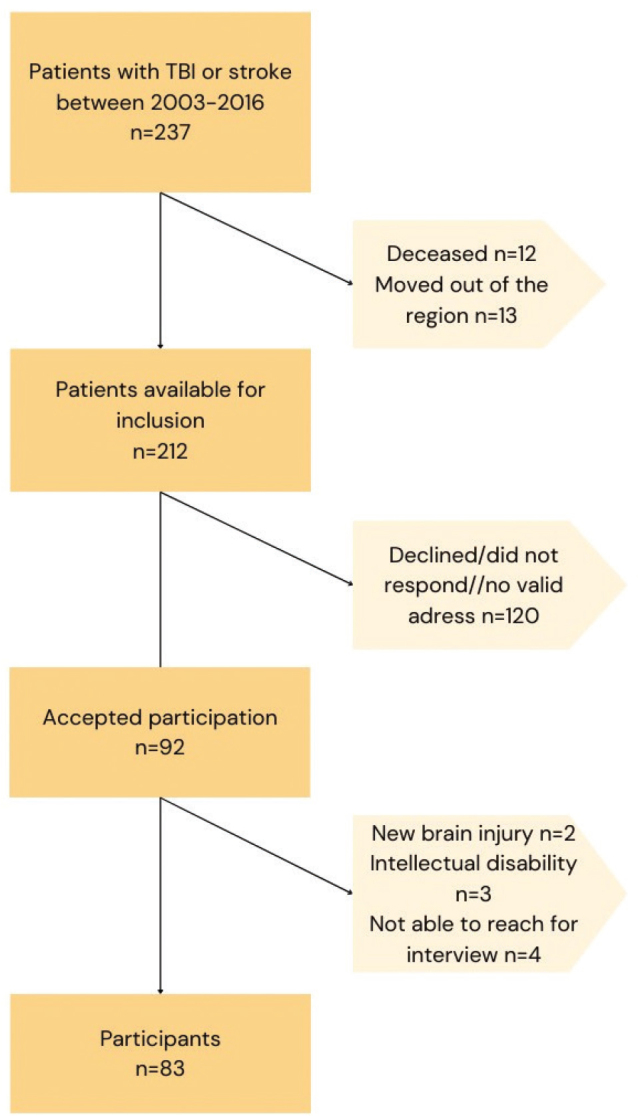
Flowchart of participant inclusion.

In total, 237 former patients of the Brain Injury Rehabilitation Team, suffering a stroke or TBI between 2003 and 2016, were eligible for participation. Mail invitations were sent to 212 patients, as 25 former patients were deceased or had moved out of the healthcare service area. Of the 92 patients willing to participate in the study, 9 were excluded. Exclusion criteria were: an intellectual disability (IQ < 70), new brain injury or being unable to be reached for an interview despite multiple attempts. In total, 83 patients were included in the study. For flowchart see Fig. 1.

## Procedure

After obtaining written consent, a structured telephone interview was scheduled with each patient. Conducted on average 9 years after the brain injury (ranging from 5 to 15 years), the interview followed a standardized manual covering employment, life satisfaction (Life Satisfaction Questionnaire LiSat-11 [17]), anxiety and depression (Hospital Anxiety and Depression Scale, HADS [18]), and general outcomes (Glasgow Outcome Scale-Extended, GOSE [19]). Present fatigue levels were self-rated by the patient on a scale from 0 to 10. Each 30-min interview was conducted by a social worker, psychologist, or psychology student with no prior relationship to the patient.

Educational level was used as a proxy for cognitive reserve, obtained through interviews and chart reviews. Data on consciousness levels at the time of injury, according to the Glasgow Coma Scale (GCS [20]) or Reaction Level Scale (RLS [21]), were collected from medical charts, with RLS scores converted to GCS for consistency (22, 23). Other diagnosis and complications at time of injury were collected from patient charts. A physician specialized in rehabilitation medicine graded the diagnoses and complications on a 3-point severity scale. For complications, 1 indicated no or mild complications (e.g., fractures not requiring medical attention, urinary tract infections), 2 indicated moderate complications (e.g., seizures, fractures requiring medical attention), and 3 indicated severe complications (e.g., heart failure, multiple fractures). For concurrent diagnoses, 1 represented none or diagnoses with minor impact on rehabilitation (e.g., allergies), 2 represented diagnoses with moderate impact on rehabilitation (e.g., high blood pressure, panic disorder), and 3 represented multiple diagnoses with at least moderate impact on rehabilitation (e.g., high blood pressure and diabetes). Prior contact with specialized psychiatry was collected through the healthcare usage data to control for previous psychiatric history.

Healthcare usage data were collected from databases, covering primary, inpatient, and outpatient specialized care, from the calendar year 3 years before the injury to the calendar year 4 years after. An 8-year span allowed for a comprehensive analysis of long-term trends and patterns in healthcare usage. The calendar year, instead of year based on date of injury, was chosen as it was not feasible to export the healthcare data on any other basis. To correct for this, the date when patients had acquired the injury was also recorded, as this is relevant for how much healthcare will be used for that calendar year. For inpatient care, number of hospital days was reported. For primary and outpatient care, number of patient-caregiver contacts was reported.

## Patient outcome variables

Return-to-work and life satisfaction were used as outcomes. Return-to-work was categorized in 2 groups, having returned at least 25% was considered as “return-to-work”, while not having returned or returned less than 25% were labelled “not return-to-work”. For patients who had reached retirement age at the time of the interview, work ability was assessed based on their pre-retirement work history. All items in LiSat-11 are validated for independent use and the first item in LiSat-11, “Satisfaction with life as a whole”, has been shown to reflect overall life satisfaction in TBI patients (24). The item can be dichotomized according to scores above 5 indicating satisfaction with life, while scores between 1 and 4 indicate dissatisfaction (25).

## Statistical analysis

All data analysis was conducted using RStudio, version 4.2.2 (31) (R Foundation for Statistical Computing, Vienna, Austria). A significance level of 0.05 was chosen for all statistical tests. Descriptive statistics were calculated using the χ^2^ test, Wilcoxon rank-sum test, and Fisher’s exact test, depending on variable measurement level (e.g., nominal, ordinal, or interval). Length of education was used as a proxy measure of cognitive reserve and categorized as low ( < 12 years) or high (≥12 years) in the ANOVA. The point of dichotomization was selected to create roughly equal groups. Additionally, in the Swedish school system, fewer than 12 years of education generally indicates a vocational or hands-on occupation, while 12 years or more indicates a professional or office-based role. Return-to-work and life satisfaction were dichotomized as mentioned earlier.

A two-way ANOVA with repeated measures (8 time points) was conducted to examine how the pattern of healthcare usage changed over time and to evaluate whether the impact of education on healthcare utilization differed before and after a brain injury. This analysis was performed for both total healthcare usage and each subtype of healthcare (inpatient, outpatient, and primary care). As the outcome variable contained outliers, the ANOVA was performed both with and without outliers, as a sensitivity analysis. Outliers were defined as total healthcare consumption above 150, which excluded 5 cases in the sensitivity analyses. Only results that remained significant when excluding outliers are reported. As the assumption of sphericity was violated, the Greenhouse–Geisser correction was applied, and the reported *p*-values are based on this correction. Additionally, although normality was violated, the within-subjects design paired with a sample size of 83 were considered sufficient to mitigate the impact of this violation.

Generalized eta squared (η²) was used to report effect sizes for all significant effects. Following Cohen’s guidelines, η² values of 0.01, 0.06, and 0.14 are interpreted as small, medium, and large effects, respectively. Estimated marginal means and their corresponding 95% confidence intervals (CI) are also presented.

A linear regression model was fitted using the “lm” function in R to investigate which variables were related to healthcare consumption injury year. Years of education, age at time of injury, diagnosis, gender, and GCS score were added to the regression model based on their potential relevance for healthcare utilization. Region of living area was initially considered for inclusion in the analysis but was excluded as it showed no significant relationship with healthcare usage in our sample. Injury date was included as healthcare usage data were collected based on the calendar year. The only missing data were in GCS and participants without GCS score were omitted from the linear regression.

Assumptions of linear regression were evaluated using diagnostic plots, including residuals vs fitted values, Q–Q plots, and scale-location plots. Visual inspection suggested that assumptions of linearity, homoscedasticity, and normality of residuals were reasonably met. Potential outliers were assessed using Cook’s distance, and no outliers were identified. Multicollinearity was assessed using variance inflation factors (VIF), and all variables had VIF values below 2, indicating low multicollinearity.

## Results

Among the 237 eligible patients, 83 completed interviews and were included in the study. No significant gender, diagnosis, or time since injury differences were found between responders and non-responders. Responders (mean age = 45 years, SD = 12) were significantly older at the time of injury than non-responders (mean age = 40 years, SD = 13), t(235) = 2.79, *p* = 0.006.

No significant differences in patient characteristics based on educational level were found (see Table I).

**Table I:** Description of patient characteristics

Item	Total, *n* = 83	Max 11 years of education, *n* = 45	12 or more years of education, *n* = 38	*p*-value
Age at time of injury, mean (SD)	45(12)	46 (12)	44 (13)	0.7
Time from injury to first contact with Outpatient Brain Injury Rehabilitation Team, median (IQR^†^)	294 (190, 504)	317 (188, 545)	276 (195, 396)	0.6
Work status				0.11
Not working, *n* (%)	45 (54%)	28 (62%)	17 (45%)	
Working at least 25%, *n* (%)	38 (46%)	17 (38%)	21 (55%)	
Gender				0.4
Female, *n* (%)	33 (40%)	16 (36%)	17 (45%)	
Diagnosis				0.3
Stroke, *n* (%)	47 (57%)	28 (62%)	19 (50%)	
TBI*, *n* (%)	36 (43%)	17 (38%)	19 (50%)	
Injury date, mean (SD)	181 (104)	192 (110)	168 (96)	0.2
Patients using specialized psychiatric healthcare in the 3-year period before the brain injury, *n* (%)	5 (6.0%)	3 (6.7%)	2 (5.3%)	> 0.9
Present levels of fatigue median (IQR^†^)	5 (4,7)	5 (3,7)	5 (4,7)	0.7
Glasgow Coma Scale, *n* (%)				0.2
3–8	16 (21%)	9 (21%)	7 (19%)	
9–12	15 (19%)	5 (12%)	10 (28%)	
13–15	47 (60%)	28 (67%)	19 (53%)	
Missing	5	3	2	
Glasgow Outcome Scale – extended, *n* (%)				0.10
Unfavourable outcome (1–5)	41 (49%)	26 (58%)	15 (39%)	
Favourable outcome (6–8)	42 (51%)	19 (42%)	23 (61%)	
Satisfied with life, *n* (%) (Score on satisfaction with life as a whole 5–6)	37(45%)	20 (44%)	17 (45%)	> 0.9
Grading of other diagnoses and complications at time of injury, median (IQR^†^)	4 (3,4)	4 (3, 4)	4 (3, 4)	0.5

SD: standard deviation; IQR: interquartile range; TBI: traumatic brain injury.

### Differences in healthcare utilization over time between educational groups

According to repeated measures ANOVA, there was a statistically significant interaction between education and time on healthcare usage (F(7,648) = 4.17, *p* = 0.008, η² = 0.035). Post-hoc tests revealed that the difference between educational level was significant during the injury year (F(1,81) = –5.47, *p* = 0.022). The high education group had an average of 77.79 hospital days/caregiver contacts (95% CI: 36.8–59.35) year of injury, compared with 55.11 (95% CI: 42.06–68.16) in the low education group.

**Fig. 2:**
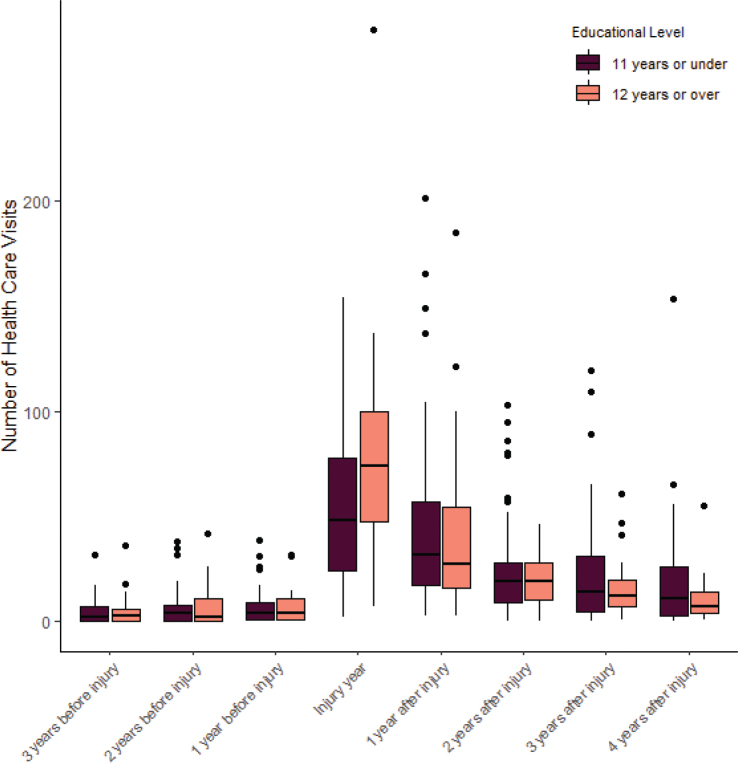
Number of healthcare visits from before to after injury separated based on years of education.

For an overview of the distribution of healthcare visits between educational groups over time, see Fig. 2.

### Differences in types of healthcare

**Fig. 3:**
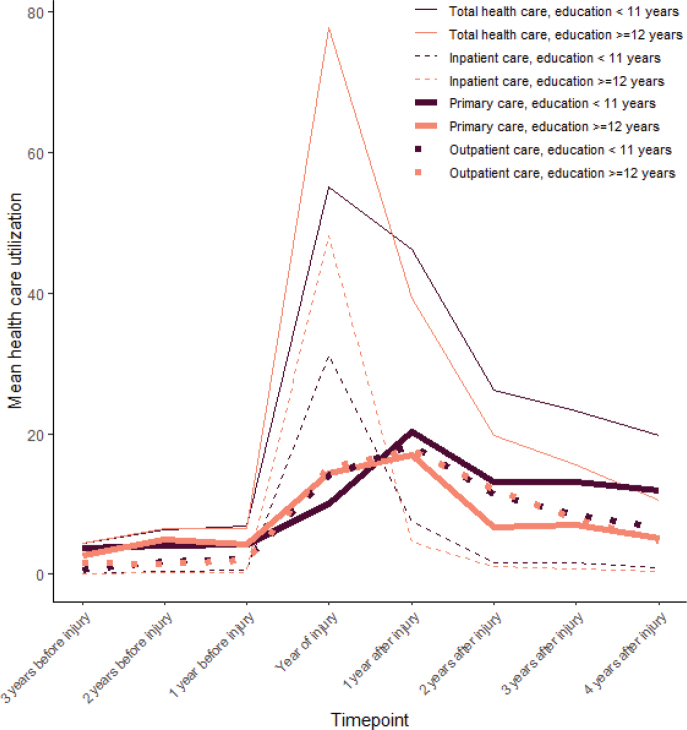
Healthcare utilization separated between different types of healthcare and levels of education. Healthcare utilization is number of days in hospital for inpatient care and number of contacts for outpatient care and primary care.

Repeated measures ANOVA found a significant interaction between time and educational level for inpatient care (F(7, 648) = 4.38, *p* = 0.045, η² = 0.045). The difference between the years was significant during the injury year (F(1,81) = –4.93, *p* = 0.029). The high education group had an average of 48.11 days in hospital (95% CI: 36.86–59.35), compared with 31.07 days (95% CI: 20.73–41.40) in the low education group. For outpatient care and primary care, there was no significant interaction between educational level and time. For a graphical overview of how healthcare utilization differed between types of healthcare over time see Fig. 3.

### Diagnosis

As the patient sample consisted of both stroke and TBI patients, a repeated-measures ANOVA was performed to investigate whether diagnosis influenced healthcare usage over time. Stroke patients used more healthcare in general F(1,81) = 8.802, *p =* 0.004, η² = 0.029. The differences in use of healthcare between diagnoses were significant 3 years before injury (F(1,81) = 4.43, *p =* 0.038), and 1 year after injury (F(1,81) = 6.32 *p =* 0.014). Participants with stroke had an average of 5.62 healthcare contacts (95% CI: 3.83–7.40) 3 years before injury, compared with 2.75 healthcare contacts (95% CI: 0.71–4.79) in the TBI group. One year after injury stroke patients had an average of 52.74 healthcare contacts (95% CI: 41.08–64.41) and TBI patients had on average 30.36 (95 CI: 17.03–43.69). No interaction between diagnosis and time point was found.

### Variables related to healthcare consumption year of injury

The linear regression model revealed that the relationship with years of education and healthcare utilization was significant even when controlling for possible confounders (see Table II). The overall regression model was also statistically significant (adjusted R^2^ = 0.15, F(6, 71) = 3.27, *p* = 0.007).

**Table II:** Linear regression investigating the relationship between education and healthcare usage

Item	Univariate	Multivariate
	Beta (95% CI†, *p*-value)	Beta (95% CI†, *p*-value)
Years of education	6.4 (2.9, 9.9, *p* < 0.001)	4.3 (0.65, 8.0, *p* = 0.022)
GCS score	–2.1 (–4.9, 0.64, 0.13)	–3.2 (–6.2, –0.13, *p* = 0.041)
Age at time of injury	0.23 (–0.58, 1.0, p = 0.6)	0.05 (–0.79, 0.89, p > 0.9)
Diagnosis – TBI*	–14 (–34, 5.4, p = 0.2)	–21 (–44, 1.6, p = 0.069)
Gender – female	2.6 (–18, 23, p = 0.8)	3.6 (–17, 24, p = 0.7)
Time (day of year) of injury	–0.11 (0.21, –0.02, *p* = 0.016)	–0.08 (–0.18, 0.01, *p* = 0.083)

GCS: Glasgow Coma Scale; TBI: traumatic brain injury.

### Correlations between healthcare utilization and return to work and life satisfaction

**Fig. 4:**
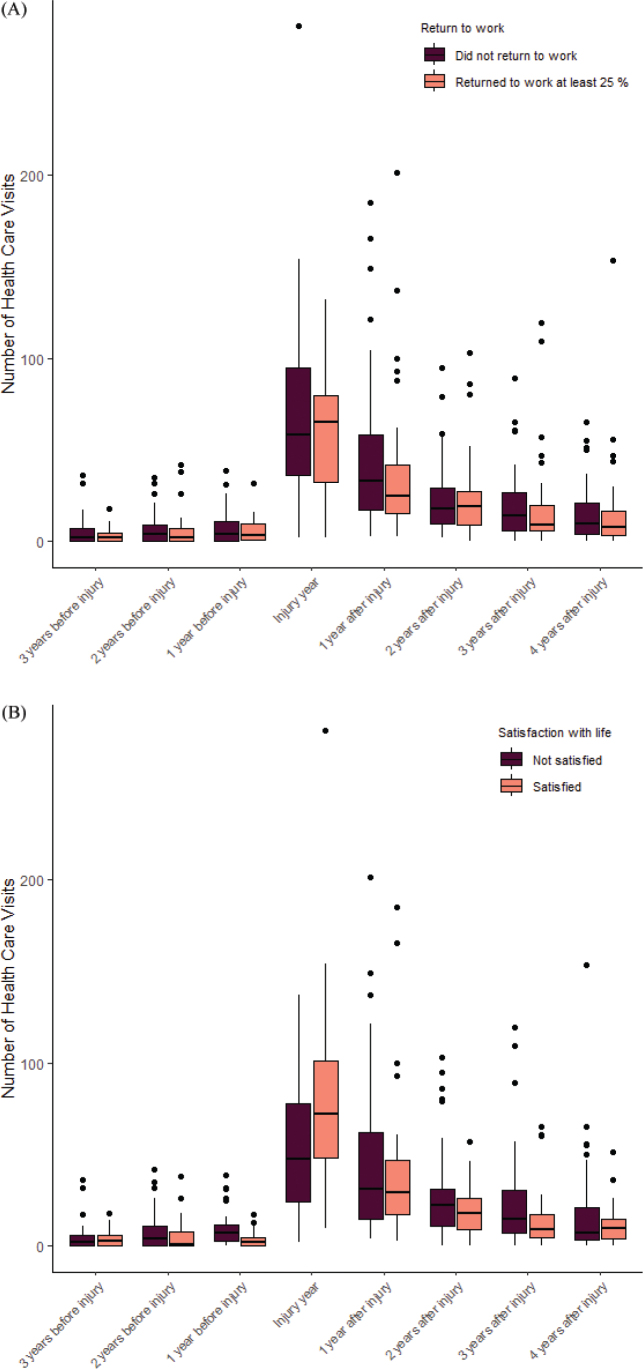
Number of healthcare visits from before to after injury separated based on (A) return to work and (B) satisfaction with life as a whole.

Return to work was not related to levels of healthcare usage (F(1, 81) = 0.791, *p* = 0.38) and there was no interaction effect between levels of healthcare usage at different time points and how that related to return to work (F(7, 648) = 0.544, *p* = 0.64) (see Fig. 4A). However, we found an interaction between time and healthcare utilization for overall life satisfaction according to the first item in LiSat-11 (F(7, 648) = 4.217, *p* = 0.008, η² = 0.035) (see Fig. 4B). The year before injury, healthcare usage was significantly higher among patients not satisfied with life 5–15 years after injury (F(1,81) = 12.5, *p* < 0.001), average healthcare usage not satisfied 6.91 (95% CI: 4.45–9.37), average healthcare usage satisfied 6.55 (95% CI: 3.88–9.23). In the injury year, this relationship reversed, and healthcare utilization was significantly higher among patients satisfied with life (F(1,81) = 5.46, *p* = 0.022), average healthcare usage not satisfied 55.11 (95% CI: 42.06–68.16), average healthcare usage satisfied 77.79 (95% CI: 63.59–91.99). Two years after injury the relationship reversed again, higher healthcare utilization was related to lower levels of life satisfaction (F(1,81) = 4.59, *p* < 0.001), average healthcare usage not satisfied 26.20 (95% CI: 20.00–32.40), average healthcare usage satisfied 19.84 (95% CI: 13.09–26.59).

## DISCUSSION

We found disparities in healthcare utilization following brain injury based on educational level. Individuals with higher education used more health services, especially inpatient care, in the injury year. This difference could not be attributed to age, diagnosis, gender, or injury severity. However, effect sizes were small, suggesting other uncontrolled factors also playing a role.

Our findings align with previous research. Adjusting for healthcare needs, individuals with higher education use more specialized care, while those with lower education rely more on primary care (3, 4, 6). However, unlike previous studies, individuals with lower education did not consume more inpatient days (26). More hospital days for people with less education have been attributed to reduced use of preventive healthcare and greater reliance on emergency visits, leading to more frequent emergency hospitalizations (27). Our study focused on patients in need of specialized rehabilitation, hence these differences in health-seeking behaviour may not apply. Previous studies have also primarily been from countries without highly subsidized healthcare, where people with less education might delay healthcare seeking.

Disparities in healthcare utilization may be linked to educational level and health literacy (28). Individuals with lower education and/or limited health literacy may not recognize the importance of timely medical attention after brain injury, resulting in treatment delays and missed opportunities for medical and rehabilitation interventions. They might also struggle to describe symptoms and advocate for care, potentially causing premature discharge.

Health literacy also affects adherence to non-medical treatments, such as physiotherapy and occupational therapy, which is common in rehabilitation medicine (29). Lower adherence to treatment would imply that extended inpatient rehabilitation is not warranted. Inpatient brain injury rehabilitation, with schedules and sessions involving various practitioners, resembles a school setting. This framework might be more appealing to individuals who have completed more years of schooling. A more flexible approach might enhance comfort for other patient groups as well.

We found no link between increased healthcare utilization during the year of injury and rates of return to work. However, more healthcare utilization during injury year was linked to life satisfaction 5–15 years after injury. This finding is unexpected, because more healthcare utilization typically correlates with more severe injuries. Interestingly, the year before injury the relationship was inverse: more healthcare utilization was related to lower life satisfaction 5–15 years later.

The finding that pre-existing circumstances, at least in part, influenced life satisfaction after brain injury is in line with previous research suggesting that non-brain-injury-related factors influence life satisfaction after brain injury (30, 31). One possible explanation for the relationship between higher life satisfaction and more healthcare during the year of injury could be the moderating effect of education. Higher education has been associated with higher rates of life satisfaction and these patients also received more healthcare (25). However, in our study we did not find a direct relationship between years of education and life satisfaction.

Our study included both stroke and TBI patients. As these are different diagnoses, variations in healthcare usage based on diagnosis can be expected. In our study stroke patients used more healthcare although not during the injury year. Diagnosis was related to healthcare usage 1 and 4 years after the injury and 3 years before injury. The lack of difference in the years closest before injury between TBI and stroke might be due to our relatively young cohort; potential prior health issues in the stroke patients might not yet have been noticed. In addition, a large study found that increased prior healthcare usage corresponded with more severe consequences after TBI (32). As all TBI patients in our study were referred for specialist rehabilitation after injury, they likely had more severe consequences from the TBI, suggesting increased healthcare use before injury.

Previous research shows that early rehabilitation is beneficial in both TBI and stroke (33, 34). Given the critical role of early rehabilitation, it is vital to further investigate the divergent patterns of healthcare use that emerged in our study. If individuals with higher education have more access to specialized healthcare during the initial phases of rehabilitation, they receive better support early on, leading to improved prospects for positive outcomes. Consequently, some of the consistent outcome differences after brain injury, which have been attributed to cognitive reserve, may partially stem from disparities in healthcare utilization.

The study had a high rate of non-responders, who were older than responders. Age might affect healthcare usage and the same differences might not be reflected in younger patient groups. Furthermore, due to limited data on non-responders, there may be other unaccounted differences affecting the generalizability of our results. The patients were interviewed 5 to 15 years after brain injury, and although outcomes after brain injury are generally considered stable after 5 years, outcomes such as life satisfaction and return to work might vary over this time period. Furthermore, other health conditions besides the brain injury might influence healthcare utilization. To control for this, additional diagnoses and complications at the time of injury were recorded and no differences between the educational groups were found. A major limitation of the study is the risk of inflated Type I error rates due to the number of statistical tests performed in the study. However, some of these tests were conducted to examine the same underlying patterns of healthcare usage from different perspectives, e.g., first overall healthcare and then looking at specific subgroups of healthcare usage to investigate where this difference in healthcare usage between groups stemmed from. Nonetheless, there is an increased risk that some of the statistically significant findings in the study are due to chance, which advises cautious interpretation. Additionally, a limitation of our study is the selective patient group. Although this group reflects an actual clinical population, it stems from a small geographical area in Sweden, though similar findings in a larger Swedish study, investigating access to rehabilitation, suggest broader applicability (16). The interaction effect between educational level and timepoint on healthcare usage in our study is considered small to medium, suggesting that factors other than education also influence healthcare usage, as expected. However, even after adjusting for other potential influencing variables, a significant relationship between education and healthcare usage remained.

In conclusion, individuals with higher education received more healthcare following a TBI or stroke. Greater healthcare use at the time of injury was associated with better life satisfaction post-injury, though this may reflect shared underlying factors. Our findings suggest that the better outcomes often linked to cognitive reserve may partly reflect unequal access to care. Clinicians should be aware that healthcare usage is influenced by factors beyond injury severity. However, due to the number of statistical tests performed, results should be interpreted with some caution, as this increases the risk of chance findings.
